# Cannabinoid Receptors and the Endocannabinoid System: Signaling and Function in the Central Nervous System

**DOI:** 10.3390/ijms19030833

**Published:** 2018-03-13

**Authors:** Shenglong Zou, Ujendra Kumar

**Affiliations:** Faculty of Pharmaceutical Sciences, The University of British Columbia, Vancouver, BC V6T 1Z4, Canada; zoeslong@gmail.com

**Keywords:** cannabinoid, endocannabinoid, receptor, signaling, central nervous system

## Abstract

The biological effects of cannabinoids, the major constituents of the ancient medicinal plant *Cannabis sativa* (marijuana) are mediated by two members of the G-protein coupled receptor family, cannabinoid receptors 1 (CB1R) and 2. The CB1R is the prominent subtype in the central nervous system (CNS) and has drawn great attention as a potential therapeutic avenue in several pathological conditions, including neuropsychological disorders and neurodegenerative diseases. Furthermore, cannabinoids also modulate signal transduction pathways and exert profound effects at peripheral sites. Although cannabinoids have therapeutic potential, their psychoactive effects have largely limited their use in clinical practice. In this review, we briefly summarized our knowledge of cannabinoids and the endocannabinoid system, focusing on the CB1R and the CNS, with emphasis on recent breakthroughs in the field. We aim to define several potential roles of cannabinoid receptors in the modulation of signaling pathways and in association with several pathophysiological conditions. We believe that the therapeutic significance of cannabinoids is masked by the adverse effects and here alternative strategies are discussed to take therapeutic advantage of cannabinoids.

## 1. Introduction

The plant *Cannabis sativa*, better known as marijuana, has long been used for medical purpose throughout human history. The first record can be traced back to ancient China around 5000 years ago, where extracts of the plant were used for relief of cramps and pain [1]. The widely-documented uses of marijuana include anti-nociception, anti-inflammation, anticonvulsant, anti-emetic, as well as recreational use, which has largely limited its medical application [1,2]. Not until half a century ago, the first light was shed on the myth of the versatility of marijuana by the discovery of Δ^9^-tetrahydrocannabinol (THC), the main psychoactive component of approximately 70 phytocannabinoids identified in the plant [3,4]. This milestone discovery led to the generation of a variety of synthetic cannabinoids with similar or distinct structures to phytocannabinoids, which finally led to the identification and successful cloning of the cannabinoid receptor 1 (CB1R) [5,6,7]. Not long after that, another cannabinoid receptor (CBR) was identified and cloned, later termed as the cannabinoid receptor 2 (CB2R) [8]. Despite only CB1R and CB2R are widely-acknowledged as CBRs, several other receptors, ranging from other G protein-coupled receptors (GPCRs) to ion channel and nuclear receptors, have been reported to interact with cannabinoids [9,10]. Meanwhile, *N*-arachidonoyl-ethanolamine (AEA; anandamide) and 2-arachidonoylglycerol (2-AG) have been discovered to serve as endogenous agonists of CBRs, namely endocannabinoids [11,12,13]. These two compounds are the first to be identified and remain the best-studied endocannabinoids, which are both derivatives of arachidonic acid [3]. In recent years, much attention has been drawn to utilizing marijuana extracts in medicine [14]. Due to the clinical application of marijuana and the non-psychoactive nature of most phytocannabinoids except THC, the therapeutic potential of these compounds has been greatly appreciated [14]. Although this area of research is quite controversial and debatable, several phytocannabinoids, especially cannabidiol (CBD), have been suggested to exert beneficial effects in various pathological conditions, including inflammation, cancer, addiction and epilepsy [14,15,16,17].

## 2. Cannabinoid Receptors

Due to the lipophilic nature of cannabinoids, it was initially thought that these compounds exert various biological effects by disrupting the cell membrane nonspecifically. However, following the discovery of THC and subsequent emerging of several chemically synthesized cannabinoids, the successful mapping and the pharmacological characterization of cannabinoid binding sites in the brain revealed the existence of a putative CBR and its similarity to GPCR nature, which was matched with the properties of an orphan GPCR that is now known as CB1R [4,5,6,7,18].

CB1R is encoded by the gene *CNR1* and consists of 472 amino acids in humans (473 amino acids in rat and mouse, with 97–99% amino acid sequence identity among these species). Several variations of *CNR1* have been associated with *Cannabis* dependence [19,20,21]. Two recent studies have described the crystal structure of the antagonist-bound CB1R independently [22,23]. A study published earlier this year described the structural changes of CB1R upon agonist binding, unraveling the conformational mechanism of the well-known diverse structures and signaling bias of CB1R agonists [24]. In addition to the canonical long form of the CB1R, two additional isoforms with shorter N-terminus have been reported, both resulting from alternative splicing [25,26]. Recently, the different expression patterns of these three isoforms have been characterized at the mRNA level in human brain, skeletal muscle, liver, and pancreatic islet [27]. The full-length CB1R dominates in the brain and skeletal muscle, whereas the CB1Rb (with 33 amino acid deletion at the N-terminus) shows a higher expression level in the liver and pancreatic islet cells where it is involved in metabolism [27]. The pharmacological and physiological properties of the two splice variants have yet to be explored, as current studies accomplished in non-human models revealed discrepancies [25,28,29].

CB2R is encoded by the gene *CNR2*, which consists of 360 amino acid in humans. It shares only 44% sequence homology with CB1R at the protein level. The CB2R also has greater species differences among humans and rodents in comparison to CB1R, as the amino acid sequence homology is slightly above 80% between humans and rodents [30,31]. In humans, two isoforms of the CB2R have been identified, with one predominantly expressed in testis and at lower levels in brain reward regions, whereas the other is mainly expressed in the spleen and at lower levels in the brain [31]. The testis isoform has a promoter that is 45 kb upstream from the spleen isoform [31]. Thus far, four rat CB2R isoforms and two mouse isoforms have been discovered [30,31].

## 3. Endocannabinoid System

The successful identification and cloning of the CB1R prompted the discovery of its first endogenous agonist, AEA, in 1992 [13]. The fact that AEA cannot fully reproduce the effects induced by THC leads to the discovery of another important endocannabinoid, 2-AG [11,12]. Most studies on the endocannabinoid system focus on these two endocannabinoids, despite the existence of the recently identified CB1R-interacting peptides and a series of arachidonic acid derivatives that generate endocannabinoid-like effects [32]. These two well-documented endocannabinoids, as pharmacologically characterized, possess distinct properties. AEA turns out to be a high-affinity, partial agonist of CB1R, and almost inactive at CB2R; whereas 2-AG acts as a full agonist at both CBRs with moderate-to-low affinity [7,32]. Interestingly, both AEA and 2-AG have been reported to interact with various receptors. Among those, the transient receptor potential cation channel subfamily V member 1 (TRPV1), which is activated by AEA, is the best-documented for its significant role in synaptic transmission and pain regulation, whereas the interaction of 2-AG and non-CBRs has emerged only recently [32]. Although AEA and 2-AG have significant differences in receptor selectivity, both endocannabinoids are produced on demand (although controversy exists in the case of 2-AG), in response to increased intracellular Ca^2+^ concentration [9,33,34]. However, AEA and 2-AG are synthesized, transported and inactivated in respective target tissues differently. In brief, AEA is catalyzed from *N*-acyl-phosphatidylethanolamine (NAPE) by NAPE-specific phospholipase D (NAPE-PLD) or via other routes not involving NAPE-PLD [3]. On the other hand, 2-AG is produced from diacylglycerol (DAG) by either DAG lipase (DAGL) α or β, although most if not all 2-AG mediating synaptic transmission in adult brain is generated by DAGLα [35]. The rate-limiting and Ca^2+^-sensitive step in AEA and 2-AG production, however, is the formation of NAPE and DAG, which are converted from phosphatidylethanolamine by *N*-acyltransferase and phosphoinositides by phospholipase C, respectively [3,35]. After release into the intracellular space, due to their uncharged hydrophobic nature, endocannabinoids are unable to diffuse freely like other neurotransmitters. Several models have been proposed to elucidate the transport of AEA: simple diffusion driven by concentration gradients generated from enzymatic degradation, endocytosis involving caveolae/lipid rafts, through certain carrier proteins like fatty acid binding proteins and heat shock protein 70 [9]. 2-AG may share the same transport system as AEA, but it is not well understood yet [36]. Once endocannabinoids are taken up by the cells, they can be degraded through hydrolysis and/or oxidation [9]. AEA is degraded by fatty acid amide hydrolase (FAAH) into free arachidonic acid and ethanolamine, whereas 2-AG is mostly hydrolyzed by monoacylglycerol lipase (MAGL) into arachidonic acid and glycerol; several other enzymes could be involved as well [9,35,37]. Oxidation of both AEA and 2-AG could involve cyclooxygenase-2 and several lipoxygenases [38].

## 4. Endocannabinoid-Mediated Signaling

**Figure 1:**
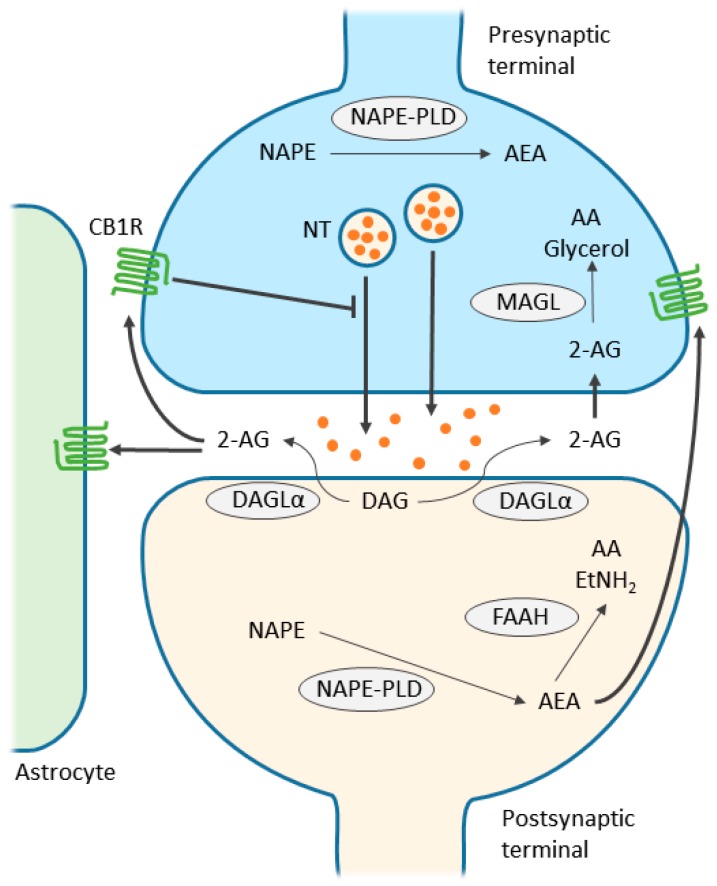
Simplified scheme representing endocannabinoid retrograde signaling mediated synaptic transmission. Endocannabinoids are produced from postsynaptic terminals upon neuronal activation. As the two major endocannabinoids shown in the scheme, 2-arachidonolglycerol (2-AG) is biosynthesized from diacylglycerol (DAG) by diacylglycerol lipase-α (DAGLα), and anandamide (AEA) is synthesized from *N*-acyl-phosphatidylethanolamine (NAPE) by NAPE-specific phospholipase D (NAPE-PLD). As lipids, endocannabinoids, mainly 2-AG, readily cross the membrane and travel in a retrograde fashion to activate CB1Rs located in the presynaptic terminals. Activated CB1Rs will then inhibit neurotransmitter (NT) release through the suppression of calcium influx. 2-AG is also able to activate CB1Rs located in astrocytes, leading to the release of glutamate. Extra 2-AG in the synaptic cleft is taken up into the presynaptic terminals, via a yet unclear mechanism, and degraded to arachidonic acid (AA) and glycerol by monoacylglycerol lipase (MAGL). On the other hand, AEA, synthesized in postsynaptic terminal, activates intracellular CB1R and other non-CBR targets, such as the transient receptor potential cation channel subfamily V member 1 (TRPV1). Although endocannabinoid retrograde signaling is mainly mediated by 2-AG, AEA can activate presynaptic CB1Rs as well. Fatty acid amide hydrolase (FAAH) is primarily found in postsynaptic terminals and is responsible for degrading AEA to AA and ethanolamine (EtNH_2_). Although NAPE-PLD is expressed in presynaptic terminals in several brain regions, it is not clear yet whether AEA is responsible for anterograde signaling in the endocannabinoid system. Note that alternative routes exist for the metabolism of endocannabinoids, depending on the brain region and physiological conditions. Thin arrows indicate enzymatic process; thick arrows indicate translocation; blunted arrow indicates inhibition.

The basal level of 2-AG is approximately 1000 times higher than AEA in the brain. Through pharmacological manipulations, altered metabolism of 2-AG, but not AEA, exerts remarkable effects in endocannabinoid-mediated retrograde signaling (Figure 1). Given these facts, it is proposed that 2-AG is the primary endogenous ligand for CBRs in the central nervous system (CNS) [32,34,35]. However, AEA has been shown to activate TRPV1, inhibit l-type Ca^2+^ channels independently, as well as negatively regulate 2-AG biosynthesis and physiological effects in striatum, underscoring its essential role in the regulation of synaptic transmission [39].

The first conclusive evidence supporting retrograde endocannabinoid signaling came from the observation of depolarization-induced suppression of inhibition (DSI)/excitation (DSE) [9,33,40]. Later, it was discovered that the endocannabinoid system is involved not only in short-term depression, but also in long-term depression (LTD) at both excitatory and inhibitory synapses [9,33]. Since then, the endocannabinoid system has become the most-studied retrograde signaling system in the brain.

In most cases, endocannabinoid-mediated retrograde signaling starts with the production of 2-AG, in response to increased intracellular Ca^2+^ concentration and/or activated G_q/11_-coupled receptors [9,33,40]. 2-AG is then released into and traverses the extracellular space, via a mechanism not yet fully elucidated, and arrives at the presynaptic terminal where it binds to the CB1R. Activated CB1R suppresses the release of neurotransmitter in two ways: first, by inhibiting voltage-gated Ca^2+^ channels, which reduce presynaptic Ca^2+^ influx; second, by inhibiting adenylyl cyclase (AC) and the subsequent cAMP/PKA pathway, which is involved in LTD [9,33,40]. The termination of signaling requires the degradation of 2-AG by MAGL, which is expressed in selective synaptic terminals and glial cells [9,33,35].

AEA has been shown to contribute to endocannabinoid-mediated synaptic transmission in several ways. AEA is a full agonist of TRPV1, which is purported to participate in endocannabinoid signaling [32]. AEA-mediated LTD (likely via a TRPV1-dependent mechanism) has been reported in several studies [41,42,43,44,45]. The differential recruitment of 2-AG and AEA by various types of presynaptic activity has been described in the extended amygdala [42]. AEA negatively regulates 2-AG metabolism, the effect of which can be mimicked by the activation of TRPV1 [39]. There is also evidence supporting a tonic role of AEA as an endocannabinoid, since chronic blockade of FAAH leads to constant agonism of the endocannabinoid system without reducing CB1R expression, which is opposite to antagonism of MAGL [46].

Endocannabinoids are prominently involved in the suppression of synaptic transmission through multiple mechanisms, independent of synaptic nature or transmission duration [9,33]. CB1R-dependent self-inhibition in postsynaptic neurons has been observed in a subpopulation of neocortical interneurons and pyramidal neurons, as well as in hippocampal CA1 neurons [47,48,49,50]. Accumulated evidence supports endocannabinoid-mediated communication between neurons and microglia [51,52,53]. Previous studies have shown that microglial cells and astrocytes are able to produce their own 2-AG or AEA, although it is not clear yet whether these endocannabinoids are involved in the modulation of synaptic transmission [54].

In contrast, although studies have shown the presence of CB2R in the brain, the role of CB2R in endocannabinoid-mediated synaptic transmission is still largely elusive [55,56,57]. A recent study has reported that in medial prefrontal cortical pyramidal neurons, intracellular CB2R reduces neuronal firing through the opening of Ca^2+^-activated chloride channels, suggesting its involvement in the regulation of neuronal activity [58].

## 5. Distribution of Cannabinoid Receptors

CB1R was first discovered in the brain. Later, by using autoradiography, in situ hybridization, and immunohistochemistry, CB1R was proven to be the most widely-expressed receptor protein from the GPCR family in the brain [9,59]. The brain regions with highest levels of CB1R expression include olfactory bulb, hippocampus, basal ganglia, and cerebellum [59]. Moderate CB1R expression is found in the cerebral cortex, septum, amygdala, hypothalamus, and parts of the brainstem and the dorsal horn of spinal cord [59]. Whereas regions like the thalamus and the ventral horn of spinal cord have low CB1R expression [59]. Several previous studies have suggested a highly concentrated expression of CB1R on presynaptic terminals, where it mediates retrograde signaling of endocannabinoids [60,61]. However, this does not preclude the existence of CB1Rs at postsynaptic sites, as functional studies demonstrate self-inhibition in neocortical neurons by endocannabinoids [33,47,49,62]. In addition to neurons, the CB1R is expressed, although to a much lower extent, in astrocytes, oligodendrocytes and microglia, where it has been shown to mediate synaptic transmission [33,54].

**Figure 2:**
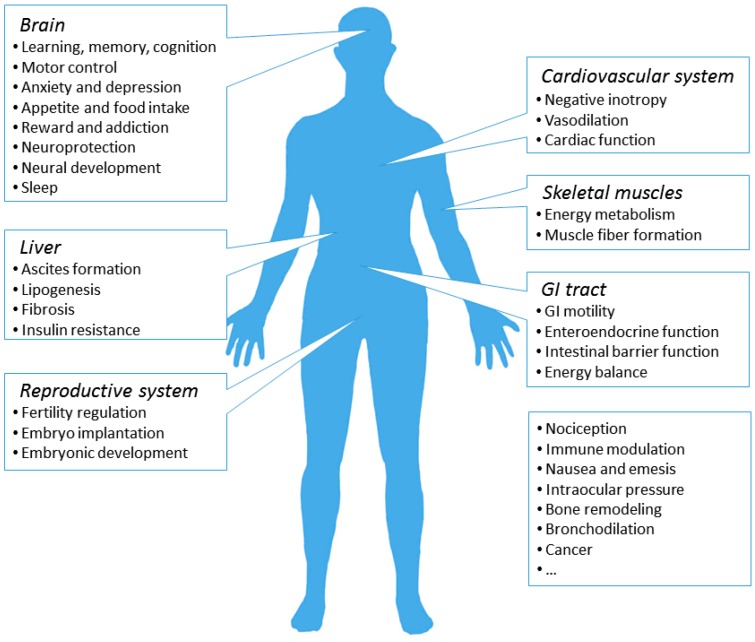
Major localization sites and associated functions of the CB1R in the human body. The majority of CB1Rs expressed in human body is found in the brain, where it is involved in various neurological activities. CB1Rs on the peripheral sites, although to a lesser extent, participates in the regulation of local tissue functions.

The CB1R is also abundantly expressed in the peripheral nervous system (PNS) as well as in the peripheral tissues in a region-specific manner [59,63,64,65] (Figure 2). In PNS, the CB1R is mostly expressed in sympathetic nerve terminals [64]. Also, the CB1R is observed in trigeminal ganglion, dorsal root ganglion, and dermic nerve endings of primary sensory neurons, where it regulates nociception from afferent nerve fibers [65,66,67]. In the gastrointestinal (GI) tract, the CB1R is enriched in both the enteric nervous system and in non-neuronal cells in the intestinal mucosa, including enteroendocrine cells, immune cells, and enterocytes [68]. Through neuronal and non-neuronal routes, the CB1R modulates the mobility of GI tract, the secretion of gastric acids, fluids, neurotransmitter and hormones, as well as the permeability of the intestinal epithelium [68]. Therefore, CB1R could control appetite from the hypothalamus in the CNS and regulate the energy balance and food intake from the GI tract as well. Intriguingly, hepatic CB1R also participates in the regulation of energy balance and metabolism, but in an unusual way. Normally, the expression of CB1R in the liver is very low [69]. However, under pathological conditions, the expression of CB1R in several types of hepatic cells is remarkably increased, where the CB1R actively contributes to hepatic insulin resistance, fibrosis, and lipogenesis [63]. Similarly, the CB1R is upregulated in the cardiovascular system under pathological conditions, which in turn, promotes disease progression and cardiac dysfunction [70]. Oxidative stress, inflammation and fibrosis have been observed as a result of CB1R activation in cardiomyocytes, vascular endothelial cells, and smooth muscle cells [70]. In addition to the aforementioned tissues, the expression of the CB1R has also been reported in adipose tissue, skeletal muscle, bone, skin, eye, reproductive system, and several types of cancer cells [63].

**Figure 3:**
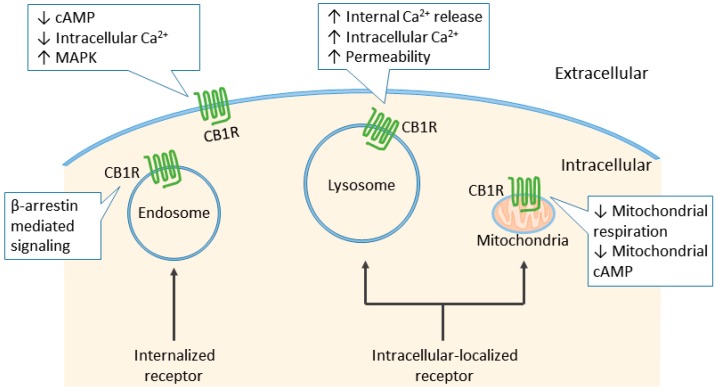
Subcellular localization of the CB1R. Typically, the CB1R is located at cell surface and inhibits cyclic adenosine monophosphate (cAMP) formation and calcium influx upon activation. Constitutive and ligand-induced internalized CB1Rs mediate signaling pathways through β-arrestin. Intracellular-localized CB1Rs do not translocate to plasma membrane. Instead, they form a subpopulation with pharmacological properties distinct from their plasma membrane-localized counterparts. CB1Rs located on lysosomes can increase intracellular calcium concentrations through the release of internal calcium stores, and increase the permeability of lysosomes. Mitochondrial CB1Rs inhibit mitochondrial cellular respiration and cAMP production, hence regulating cellular energy metabolism.

Like many other GPCRs, the CB1R is primarily localized in the cell membrane. However, besides the well-known plasma membrane localized CB1R, which is the typical distributional pattern of GPCRs, considerable observations have reported predominant intracellular localization of CB1Rs in diverse types of cells, including transfected non-neuronal cells, undifferentiated neuronal cells, and cultured hippocampal neurons [71]. Follow-up studies discovered that CB1Rs localized in intracellular compartments presumably consist of several distinct subpopulations (Figure 3). One proportion of intracellular CB1Rs comes from the continuous internalization of plasma membrane-localized CB1R [72]. Aside from the constitutive and agonist-induced internalized CB1R, accumulated evidence suggests a distinct pool of intracellularly localized CB1R, with differential functionalities from their plasma membrane-localized counterparts. These intracellular CB1Rs are in acid-filled endo/lysosomes, and do not contribute to the subpopulation expressed at the cell surface [73,74]. Moreover, the endo/lysosome-located CB1Rs increase the release of calcium from the endoplasmic reticulum and lysosomes upon activation by intracellular agonist administration [75]. Another subpopulation of CB1Rs, as suggested by several lines of evidence, is expressed in mitochondria. Previous studies have reported the effect of THC on mitochondria-associated enzymatic activity, which was attributed to the non-specific membrane disruption of lipophilic cannabinoids at that time [76]. However, recent studies have challenged this concept by demonstrating the presence of mitochondrial CB1R and its direct involvement in cellular respiration and DSI in hippocampal neurons [77]. Although there are discrepancies in the amplitude of the CB1R agonist-induced decreases in mitochondrial respiration, the existence and functionality of mitochondrial CB1Rs are undeniable [78,79,80]. Moreover, the role of mitochondrial CB1R was further expanded by several recent observations suggesting its association with cannabinoid-induced feeding behavior in hypothalamic proopiomelanocortin (POMC) neurons, memory impairment in hippocampus, and neuroprotection after cerebral ischemia/reperfusion injury [81,82,83]. These lines of evidence highlight the direct association between mitochondrial CB1R and proper functioning of mitochondria, which has been suggested to participate in many pathological conditions [84,85]. Therefore, the role of mitochondrial CB1R may not be limited to the previously discovered roles and is worth further exploration. [75,77,86].

Three years after the discovery of the CB1R, another CBR, the CB2R, was identified in macrophages in the spleen [8]. Follow-up studies revealed a predominant expression of the CB2R in immune cells and a moderate expression in other peripheral tissues, including the cardiovascular system, GI tract, liver, adipose tissue, bone, and reproductive system [10]. In contrast, the presence of the CB2R was not observed in the CNS, thus it was referred to as “the peripheral CBR” [10]. However, this concept has been challenged recently by several studies demonstrating the expression of the CB2R in the brain, albeit to a much lower extent in comparison to the immune system or the CB1R [57]. Although the expression of the CB2R in the CNS and PNS is comparatively limited, it is undeniable that the CB2R plays an active role in neurological activities, such as nociception, drug addiction and neuroinflammation [55,56]. Moreover, recent studies discovered the intracellular presence of CB2R in prefrontal cortical pyramidal neurons where it modulates neuronal excitability through the regulation of Ca^2+^-activated Cl^−^ channel [58]. In transfected human bone osteosarcoma epithelial cells, intracellularly located CB2R regulates Ca^2+^ in a faster and more potent way in comparison to CB2R expressed at cell surface [87].

## 6. Cannabinoid Receptor Signaling

Both the CB1R and CB2R are members of the GPCR family and are coupled to pertussis toxin (PTX)-sensitive G_i/o_ protein, suppress AC and the formation of cAMP upon receptor activation [10]. However, the CB1R but not the CB2R has been reported to activate other G proteins in certain circumstances in a cell type- and ligand-dependent manner [88]. The CB1R is able to stimulate specific AC isoforms via Gβγ subunits [89]. Also, the CB1R stimulates cAMP via coupling to G_s_ when the dopamine receptor 2 (D2R) is activated simultaneously in cultured striatal neurons, and when G_i_ is blocked by PTX in transfected CHO-K1 cells, and in response to a relatively high concentration of WIN 55, 212-2 (WIN) in rat globus pallidus slices [90,91,92]. However, the same concentration of WIN but not other CB1R agonists, increases intracellular Ca^2+^ concentration via G_q/11_ protein in transfected HEK-293 cells and cultured hippocampal neurons with endogenous receptor expression [93]. Moreover, in mice hippocampal slices, CB1R expressed in astrocytes is coupled to G_q/11_, increases intracellular Ca^2+^ concentrations and triggers astrocytic release of glutamate that stimulates *N*-methyl-d-aspartate receptor (NMDAR) on pyramidal neurons, indirectly involved in synaptic transmission [53].

Moreover, the CB1R modulates the activity of several types of ion channels [88,94]. CB1Rs have been reported to inhibit N-type Ca^2+^ channel in neuroblastoma cell lines, in cultured rat primary hippocampal neurons, and in mice cerebellar slices [95,96,97,98]. It has long been suggested, but proved only recently, that the CB1R regulates Ca^2+^ influx to inhibit γ-aminobutyric acid (GABA) release in mouse hippocampal slices via modulation of the activity of presynaptic N-type Ca^2+^ channel [99]. Other types of Ca^2+^ channels, including P/Q-type, and R-type Ca^2+^ channels, have been shown to be negatively regulated by CB1R in various systems [95,96,100,101]. On the other hand, the CB1R regulates the activity of G-protein-coupled inwardly rectifying K^+^ channels (GIRKs) as well [101,102,103]. The CB1R activates GIRK in transfected AtT-20 cells, mouse nucleus accumbens slices, and rat sympathetic neurons injected with CB1R complementary deoxyribonucleic acid (cDNA) [101,102,103].

Previous studies have shown that in a system expressing the receptor endogenously or heterogeneously, stimulation of CB1R leads to the activation of mitogen-activated protein kinase (MAPK) signaling pathways, including extracellular signal-regulated kinase 1/2 (ERK1/2), c-Jun N-terminal kinase (JNK), and p38, that are involved in the regulation of cell proliferation, cell cycle control and cell death [88,94,104]. Generally, CB1R regulates MAPK signaling in a cell type- and ligand-specific fashion [88,94,104]. For instance, CB1R-induced ERK1/2 activation can be mediated by G protein, β-arrestin, or phosphatidylinositol-3-kinases (PI3K), heavily dependent on the microenvironment and stimulus type [105,106,107]. Similarly, activation of p38 has been observed upon stimulation of CB1R in human vascular endothelial cells, transfected CHO-K1 cells, and rat/mouse hippocampal slices [108,109,110]. JNK activation has been shown in transfected CHO-K1 cells, where G proteins, PI3K and Ras were involved in the transduction [109]. Moreover, JNK activation was also observed in Neuro2A cells with endogenous expression of CB1R, and may be related to CB1R-mediated neurite outgrowth [111].

In addition to the typical G protein-dependent signaling seen with all GPCRs, the CB1R is able to signal in a G protein-independent manner through association with other molecules such as β-arrestin [104]. β-arrestin is a key mediator of GPCR desensitization. Following receptor phosphorylation by GRK, β-arrestin binds to the receptor and initiates the internalization process, during which β-arrestin could mediate signaling pathways [112]. Desensitization of the CB1R has been shown to be β-arrestin 2-dependent in various systems [113,114]. It has been reported in transfected HEK-293 cells that β-arrestin 2-mediated desensitization but not internalization of CB1R determines the time course of ERK1/2 phosphorylation upon CB1R activation [115]. Furthermore, follow-up studies revealed a positive correlation between the extent of β-arrestin-mediated signaling and the duration of CB1R interaction with β-arrestin at the cell surface in a ligand-specific manner [106]. Studies using β-arrestin 2 knockout mice have suggested a critical role of β-arrestin 2 in the regulation of CB1R activity [116,117]. The β-arrestin 2 knockout mice displayed a comparable expression level of CB1R yet an increased sensitivity to THC, featuring enhanced antinociception and decreased tolerance [116,117]. A recent study suggested a role of β-arrestin 1 in the phosphorylation of ERK1/2, MAPK kinase 1/2 and the proto-oncogene tyrosine-protein kinase Src in response to a CB1R allosteric modulator ORG27569, underscoring a signaling mechanism that is largely dependent on stimulus [118].

**Figure 4:**
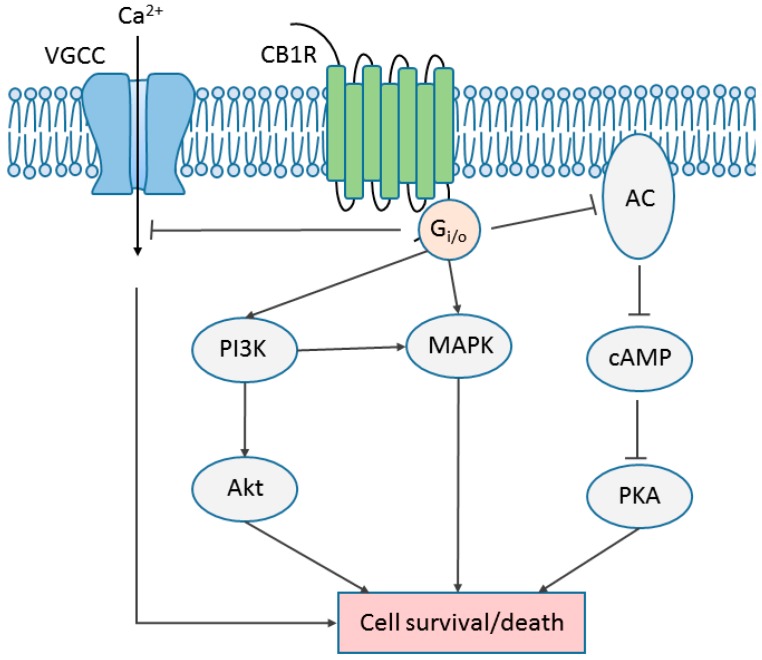
CB1R-modulated major signaling pathways. Typically, the CB1R is coupled to G_i/o_ and inhibits the activity of adenylyl cyclase (AC), formation of cyclic adenosine monophosphate (cAMP), and the activity of protein kinase A (PKA). Under certain circumstances, the CB1R can switch its coupling of G protein from G_i/o_ to G_s_ or G_q_. The CB1R is able to suppress calcium influx via voltage-gated calcium channel (VGCC). Several mitogen-activated protein kinases (MAPKs), including ERK1/2, p38, and JNK, are activated by the CB1R. The phosphoinositide 3-kinase (PI3K)/protein kinase B (Akt) pathway is activated by CB1R as well. Depending on the ligand and subcellular environment, the outcome of CB1R-mediated signaling could be promotion of cell survival or cell death. Arrows indicate stimulation; blunted arrows indicate inhibition.

The PI3K/Akt pathway is another key regulator of cell growth and death aside of MAPK signaling. In rat primary cultured astrocytes, human astrocyte cell line, and transfected CHO-K1 cells, the CB1R has been shown to activate the PI3K/Akt pathway, which is responsible for the CB1R-induced protective effects on cell survival [105,111,119]. In rat oligodendrocyte progenitors, the CB1R promotes cell survival against nutrient deprivation and modulate cell differentiation via the PI3K/Akt pathway [120,121]. Similarly, in rat cortical cultured neurons, a CB1R selective agonist, HU-210, exerts neuroprotective effects against the neurotoxin (*S*)-α-amino-3-hydroxy-5-methyl-4-isoxazolepropionic acid through activation of the PI3K/Akt pathway but not MAPK pathways [122]. A previous study in mice demonstrated that acute administration of THC activated the PI3K/Akt pathway, but not ERK1/2 in several brain regions [123]. A recent study in *huntingtin* knock-in striatal neuronal cells showed that CB1R protected neurons against excitotoxicity via PI3K/Akt signaling-mediated increase in brain-derived neurotrophic factor (BDNF) expression [124]. In addition, CB1R-mediated PI3K/Akt activation has also been shown to modulate oocyte maturation and embryonic development [125] (Figure 4).

## 7. Physiological and Pathological Roles of the CB1R

Given the widespread distribution of CB1Rs in the human body, it is reasonable for one to speculate a broad spectrum of physiological roles of the CB1R [3,9,63,126]. Indeed, the CB1R and the endocannabinoid system are largely involved in various aspects of central neural activities and disorders, including appetite, learning and memory, anxiety, depression, schizophrenia, stroke, multiple sclerosis, neurodegeneration, epilepsy, and addiction [3,9,126,127]. The CB1R is also involved in physiological and pathological conditions in the PNS and peripheral tissues, including pain, energy metabolism, cardiovascular and reproductive functions, inflammation, glaucoma, cancer, and liver and musculoskeletal disorders [63]. The expression of CB1R remarkably fluctuates in many pathological conditions, underscoring its critical role in a wide spectrum of biological activities [69]. Interestingly, in some cases, both positive and negative alterations in CB1R expression and functionality have been reported [69]. Moreover, the administration of CB1R agonists exert biphasic effects in several conditions [128]. On the other hand, the widespread presence of the CB1R limits the therapeutic application of CB1R ligands due to various side effects. These facts underscore the significance of understanding and manipulating the endocannabinoid system in a condition-specific manner.

CB1R has been found to inhibit GABA and glutamate release from presynaptic terminals, which confers the CB1R with the ability to modulate neurotransmission [60,129]. This has been proposed as a plausible underlying mechanism of CB1R-mediated neuroprotection against excitotoxicity, a prominent pathological process of many neurological disorders, including epilepsy and neurodegenerative diseases [34,130,131]. To date, numerous studies have shown that the CB1R plays a neuroprotective role against excitotoxicity induced by various stimuli [131,132,133,134]. It has been demonstrated recently that in mouse brain, the neuroprotective effect exerted by CB1R against excitotoxicity is restricted to the CB1R population located on glutamatergic terminals [130]. In addition to the prominent inhibitory effects on Ca^2+^ influx and glutamate release, CB1R-mediated neuroprotection also involves inhibition of nitric oxide (NO) production, reduction of zinc mobilization, and increase of BDNF expression [134,135,136]. Recent studies have implicated a direct physical interaction between CB1Rs and NMDARs in the presence of histidine triad nucleotide-binding protein 1, which allows CB1Rs to negatively regulate NMDAR activity and protects neural cells from excitotoxicity [136,137].

Specifically, altered expression of the CB1R and other elements of the endocannabinoid system have been observed in various neurodegenerative diseases, such as Alzheimer’s disease (AD), Parkinson’s disease (PD) and Huntington’s disease (HD) [3]. The upregulation of the CB1R and endocannabinoid system activity has been observed in the basal ganglia of experimental models of PD, which could be a mechanism to compensate the degenerated dopaminergic neurons of the substantia nigra, or a pathological process that contributes to the worsening of the disease [138]. Interestingly, decreased endocannabinoid system activity has also been reported in PD models [128]. Moreover, both the FAAH inhibitors and CB1R antagonists have been shown to alleviate the motor symptoms in PD models [128]. Similarly, although changes of CB1R expression in AD patients or animal models are still controversial, the activation of the CB1R has been shown to prevent amyloid β-induced neurotoxicity in several cell models [139,140,141,142,143,144]. In addition, the activation of the CB1R has been reported to be beneficial in AD animal models with memory deficits and cognitive disorders [145,146,147]. On the other hand, studies have emphasized the beneficial potentials of the CB1R in HD pathogenesis. In 1993, decreased expression of the CB1R was first reported in the substantia nigra of HD patients via autoradiography [148]. Further studies revealed a progressive loss of CB1Rs as an early sign of HD, which occurred before the onset of actual neurodegeneration, and hastened the worsening of HD [149]. This observation was confirmed at the mRNA level as well as with CB1R immunoreactivity in several transgenic HD mouse models (reviewed in [3]). A recent study described downregulation of the CB1R not only in medium spiny projection neurons (MSNs) but also in a subpopulation of interneurons that are selectively preserved in both transgenic HD mice and HD patients [150]. Delayed loss of CB1Rs in HD transgenic mice R6/1 was seen in enriched environment, accompanied by delayed onset of motor disorders and disease progression [151]. Moreover, in HD transgenic mice R6/2, CB1R knockout leads to the worsening of motor performances, increased susceptibility to 3-nitropropionic acid, and exacerbated striatal atrophy and Huntingtin (Htt) aggregates [133,152]. Selective increase in CB1R expression in MSNs improves the survival of excitatory projection neurons, but does not promote the motor performances of HD transgenic R6/2 mice [153]. Administration of THC has been reported to ameliorate motor disorders, striatal atrophy, and Htt aggregates in transgenic mice, although controversy exists [133,154]. Activation of the CB1R inhibits glutamate release while increases BDNF release from presynaptic terminals in mice [131]. Further investigation in HD cell models revealed that CB1R activation can protect striatal cells against excitotoxicity through increased BDNF expression via PI3K/Akt pathway [133]. These observations support a critical and possibly beneficial role of the CB1R in neurodegenerative diseases.

The historical record of the anti-epileptic effects of the CB1R dates back centuries [1]. Case reports on the beneficial effects of cannabinoids on epileptic patients became available only after the identification of THC [155,156]. However, studies also suggested increased seizure frequency after marijuana smoking [157]. This paradoxical effect of cannabinoids on epilepsy is not only seen in human studies but has also been reported in animal models [158,159]. Activation of the CB1R by AEA has been shown to inhibit electroshock-induced seizures in rats [159]. Conversely, CB1R activation in FAAH knockout mice displays increased susceptibility to kainic acid-induced seizures [158]. The alteration of the endocannabinoid system following epilepsy is cell type-specific. This concept is supported by previous animal studies showing that CB1R retrograde signaling is selectively enhanced at inhibitory but not excitatory synapses, resulting a persistent potentiation of DSI but not DSE in febrile seizures, which leads to hyper-excitability of neurons, thus contributing to the exacerbation of seizures [160,161]. Moreover, this CB1R-mediated enhanced suppression of inhibitory neurons is phase-dependent as well. Hippocampal tissues from epileptic patients in the acute phase of epilepsy display decreased CB1R density, especially in the dentate gyrus, whereas in patients in the chronic phase of epilepsy, an upregulation of CB1R has been observed [162,163,164,165].

Despite the low expression of CB1R in hypothalamus, cannabinoids are long known for their effects to stimulate appetite, prominently in a CB1R-dependent manner [166]. Endocannabinoids levels are increased in the rat hypothalamus during fasting and return to normal levels after food consumption [167]. The stimulation of appetite and feeding behavior is observed after direct injection of endocannabinoids and is abolished by the administration of CB1R antagonists [167]. Furthermore, activation of ventral striatal CB1Rs inhibit GABAergic neurons, resulting in a hypophagic but not an orexinergic effect [168]. A recent study has demonstrated that CB1R-induced feeding behavior is promoted by the activation of hypothalamic POMC neurons [81]. In addition to the hypothalamus, olfactory process have been proposed to be involved in the positive regulation of CB1R-mediated food intake [169]. Moreover, crosstalk between CB1Rs and the important hormones involved in appetite regulation, including ghrelin, leptin, and orexin, has been extensively reported [68,166]. CB1Rs expressed in the GI tract also are involved in metabolic process and energy balance, as discussed in the previous section. These studies suggest that CB1R-mediated regulation of appetite involves at least two aspects: through the regulation of CNS region related to appetite, and through the modulation of metabolic hormones and digestive functions on site. Rimonabant, a CB1R antagonist, displayed remarkable anti-obesity effects, yet the accompanying psychiatric side effects lead to its withdrawal from the market [170]. An up-to-date review by Koch have summarized the recent progress on elucidating the role of CB1R in appetite control [171].

The regulation of pain is one of the earliest medical applications of cannabinoids [1,2]. Numerous studies have documented the analgesic effects of cannabinoids in different types of pain, including chemical, mechanical, and heat pain, as well as neuropathic, inflammatory, and cancer pain [172,173]. The endocannabinoid system also is involved in the regulation of nociception [3]. A newly published review paper has discussed the preclinical and clinical studies on the role of endocannabinoids in the control of inflammatory and neuropathic pain in details [173]. In addition to the CB1R, there also is evidence supporting the involvement of the CB2R and TRPV1 in cannabinoid-mediated regulation of pain [174,175]. Furthermore, the phytocannabinoids have drawn much attention nowadays in the field of antinociception and other neurological disorders. CBD, for instance, has been shown to modulate chronic pain in several studies [173]. The drug with brand name Sativex, containing equal amount of THC and CBD, is used to treat several kinds of multiple sclerosis associated symptoms including chronic pain [176]. Despite the fact that CBD has negligible affinity to the CB1R and CB2R, recent studies have suggested that it is an allosteric modulator and an indirect antagonist of CBRs, with the ability to potentiate the effect of THC [177].

Cannabinoids used in cancer are best-known for their palliative effects, including reducing nausea and vomiting, alleviating cancer pain, and stimulating appetite [178,179]. It has been argued that cannabinoids can exert anti-tumor effects directly through the inhibition of cell proliferation and induction of apoptosis, or indirectly through the inhibition of angiogenesis, invasion and metastasis [180]. Numerous studies using synthetic/endo-/phyto-cannabinoids and endocannabinoid system regulators in various cancer cell lines support this notion [181]. The antitumor effects of cannabinoids have also been observed in various animal tumor models [180]. In general, an enhanced endocannabinoid system is seen in tumor tissues [179,182,183]. However, the role of upregulated endocannabinoid system activity is still controversial as contrasting results have been reported supporting a proliferative as well as an anti-proliferative role of cannabinoids on cancer cells [180,181]. Interestingly, a bimodal effect of cannabinoids on cancer cell growth has also been observed, with low concentrations being proliferative and high concentrations being pro-apoptotic [184].

## 8. Future Directions of Cannabinoid-Based Drug Discovery

Most cannabinoid-base drugs available now in market are THC derivatives, indicated for anorexia and emesis associated with chemotherapy [185]. As a result of systematic activation of the CB1R, the accompanying side effects always include cardiovascular dysfunction, digestion failure, neurological disorders and potential for addiction [186]. The goal of cannabinoid-based drugs is to fully explore their promising therapeutic potentials without these adverse effects and the success of Sativex provides some insights. First, phytocannabinoids may block the undesired psychoactive effects of compounds targeting CB1R. Although the exact mechanism of how a 1:1 ration of CBD to THC enables Sativex to be well-tolerated by patients is not clear, the addition of CBD certainly contributes to the prevention of the associated side effects. Second, phytocannabinoids alone possess great potential as drug targets. Excluding THC, all phytocannabinoids identified so far are non-psychoactive, making them a safer choice and a great pool for drug screening. Encouraging results have been reported on their therapeutic potential in various diseases [15,17]. Third, allosteric modulator designed to modify the effect of CB1R agonists/antagonists may be beneficial in minimizing the side effects. Research has progressed significantly towards this direction in the past few years, with several synthetic or natural compounds characterized as CB1R allosteric ligands [177,187,188,189]. A detailed review on their pharmacological properties and therapeutic potentials is available [190].

Alternative way to modulate the effect of CB1R is through heteromerization with other GPCRs [104,191]. Chimerical compounds targeting GPCR heterodimers, including delta-opioid receptor/mu-opioid receptor and somatostatin receptor 5/dopamine receptor 2, have been successfully generated and used in clinical practice [192,193,194]. CB1R has been shown to heterodimerize with several GPCRs, with distinct pharmacological properties, emphasizing its significance in different pathological conditions [104,191]. Efforts have been made to utilize these findings in drug discovery focusing on specific heterodimer complex, although recent findings on the structures of CB1R and other lipid-binding receptor suggest that the currently available bivalent ligands targeting CB1R homo- or heterodimers are unlikely to bind both protomers simultaneously [195,196]. More information on CB1R structure and dimerization interface is needed for better design of bivalent and dualsteric ligands. 

Besides CB1R, other elements in the endocannabinoid system have become targets of drug discovery as well. Inhibitors of enzymes that degrade endocannabinoids, such as FAAH inhibitors, work effectively as an alternative way of CB1R activation and endocannabinoid tone enhancement, although caution should be taken in the use of these drugs due to their potential off-target activities [197]. On the other hand, CB2R is also attracting more interest, especially on the peripheral sites, where studies have shown its beneficial effects in various pathological conditions [55]. Also, recent studies have discovered its presence and significance in the CNS, revealing another exciting therapeutic potential of CB2R [56].

## 9. Conclusions

The initial discovery and subsequent intensive research of the endocannabinoid system in the last three decades have revealed probably the most well-known retrograde neurotransmission system. As the main mediator of psychoactive effect of THC, CB1R has gained tremendous interest over these years. Its widespread expression and versatile functions not only support its promising potential as a drug target for various diseases, but also make the undesired side effects almost inevitable. This obstacle leads researchers to pay more attention to the long-ignored CB2R and other endo-/phyto-cannabinoids. Moreover, as a neuromodulator, the crosstalk between endocannabinoid and other neurotransmitter systems, via either local neural circuits, or receptor heteromerization, or downstream signaling, has been emphasized. Fruitful studies have been generated, unraveling the complexity of the whole endocannabinoid system. It is critical to keep in mind that the study of the endocannabinoid system should be region- and condition-specific, along with the consideration of other neurotransmission systems.

## Abbreviations

THC	Δ^9^-tetrahydrocannabinol
CBR	Cannabinoid receptor
AEA	*N*-arachidonoyl-ethanolamine
2-AG	2-arachidonoylglycerol
CBD	Cannabidiol
GPCR	G protein-coupled receptor
TRPV1	Transient receptor potential cation channel subfamily V member 1
NAPE	*N*-acyl-phosphatidylethanolamine
NAPE-PLD	NAPE-specific phospholipase D
DAG	Diacylglycerol
DAGL	DAG lipase
FAAH	Fatty acid amide hydrolase
MAGL	Monoacylglycerol lipase
CNS	Central nervous system
DSI	Depolarization-induced suppression of inhibition
DSE	Depolarization-induced suppression of excitation
LTD	Long-term depression
AC	Adenylyl cyclase
PNS	Peripheral nervous system
GI	Gastrointestinal
cAMP	Cyclic adenosine monophosphate
PTX	Pertussis toxin
WIN	WIN 55,212-2
NMDAR	*N*-methyl-d-aspartate receptor
cDNA	Complementary deoxyribonucleic acid
GABA	γ-aminobutyric acid
GIRK	G-protein-coupled inwardly rectifying K^+^ channel
ERK1/2	Extracellular signal-regulated kinase 1/2
JNK	c-Jun N-terminal kinase
MAPK	Mitogen-activated protein kinase
PI3K	Phosphatidylinositol-3-kinases
BDNF	Brain-derived neurotrophic factor
NO	Nitric oxide
AD	Alzheimer’s disease
PD	Parkinson’s disease
HD	Huntington’s disease
MSN	Medium spiny projection neuron
Htt	Huntingtin

## References

[1] Mechoulam R. (1986). The Pharmacohistory of Cannabis sativa, in Cannabis as Therapeutic Agent.

[2] Iversen L. (2000). The Science of Marijuana.

[3] Pacher P., Batkai S., Kunos G. (2006). The endocannabinoid system as an emerging target of pharmacotherapy. Pharmacol. Rev..

[4] Gaoni Y., Mechoulam R. (1964). Isolation, structure, and partial synthesis of an active constituent of hashish. J. Am. Chem. Soc..

[5] Matsuda L.A., Lolait S.J., Brownstein M.J., Young A.C., Bonner T.I. (1990). Structure of a cannabinoid receptor and functional expression of the cloned cdna. Nature.

[6] Devane W.A., Dysarz F.A., Johnson M.R., Melvin L.S., Howlett A.C. (1988). Determination and characterization of a cannabinoid receptor in rat brain. Mol. Pharmacol..

[7] Pertwee R.G., Howlett A.C., Abood M.E., Alexander S.P., di Marzo V., Elphick M.R., Greasley P.J., Hansen H.S., Kunos G., Mackie K. (2010). International union of basic and clinical pharmacology. LXXIX. Cannabinoid receptors and their ligands: Beyond CB1and CB_2_. Pharmacol. Rev..

[8] Munro S., Thomas K.L., Abu-Shaar M. (1993). Molecular characterization of a peripheral receptor for cannabinoids. Nature.

[9] Kano M., Ohno-Shosaku T., Hashimotodani Y., Uchigashima M., Watanabe M. (2009). Endocannabinoid-mediated control of synaptic transmission. Physiol. Rev..

[10] Howlett A.C., Barth F., Bonner T.I., Cabral G., Casellas P., Devane W.A., Felder C.C., Herkenham M., Mackie K., Martin B.R. (2002). International union of pharmacology. XXVII. Classification of cannabinoid receptors. Pharmacol. Rev..

[11] Sugiura T., Kondo S., Sukagawa A., Nakane S., Shinoda A., Itoh K., Yamashita A., Waku K. (1995). 2-arachidonoylgylcerol—A possible endogenous cannabinoid receptor-ligand in brain. Biochem. Biophys. Res. Commun..

[12] Mechoulam R., Benshabat S., Hanus L., Ligumsky M., Kaminski N.E., Schatz A.R., Gopher A., Almog S., Martin B.R., Compton D.R. (1995). Identification of an endogenous 2-monoglyceride, present in canine gut, that binds to cannabinoid receptors. Biochem. Pharmacol..

[13] Devane W.A., Hanus L., Breuer A., Pertwee R.G., Stevenson L.A., Griffin G., Gibson D., Mandelbaum A., Etinger A., Mechoulam R. (1992). Isolation and structure of a brain constituent that binds to the cannabinoid receptor. Science.

[14] Izzo A.A., Borrelli F., Capasso R., di Marzo V., Mechoulam R. (2009). Non-psychotropic plant cannabinoids: New therapeutic opportunities from an ancient herb. Trends Pharmacol. Sci..

[15] Hill A.J., Williams C.M., Whalley B.J., Stephens G.J. (2012). Phytocannabinoids as novel therapeutic agents in cns disorders. Pharmacol. Ther..

[16] Mechoulam R., Sumariwalla P.F., Feldmann M., Gallily R. (2005). Cannabinoids in models of chronic inflammatory conditions. Phytochem. Rev..

[17] Patil K.R., Goyal S.N., Sharma C., Patil C.R., Ojha S. (2015). Phytocannabinoids for cancer therapeutics: Recent updates and future prospects. Curr. Med. Chem..

[18] Pertwee R.G. (2006). Cannabinoid pharmacology: The first 66 years. Br. J. Pharmacol..

[19] Schacht J.P., Hutchison K.E., Filbey F.M. (2012). Associations between cannabinoid receptor-1 (CNR1) variation and hippocampus and amygdala volumes in heavy cannabis users. Neuropsychopharmacology.

[20] Hartman C.A., Hopfer C.J., Haberstick B., Rhee S.H., Crowley T.J., Corley R.P., Hewitt J.K., Ehringer M.A. (2009). The association between cannabinoid receptor 1 gene (CNR1) and cannabis dependence symptoms in adolescents and young adults. Drug Alcohol Depend..

[21] Agrawal A., Lynskey M.T. (2009). Candidate genes for cannabis use disorders: Findings, challenges and directions. Addiction.

[22] Hua T., Vemuri K., Pu M., Qu L., Han G.W., Wu Y., Zhao S., Shui W., Li S., Korde A. (2016). Crystal structure of the human cannabinoid receptor CB1. Cell.

[23] Shao Z., Yin J., Chapman K., Grzemska M., Clark L., Wang J., Rosenbaum D.M. (2016). High-resolution crystal structure of the human CB1cannabinoid receptor. Nature.

[24] Hua T., Vemuri K., Nikas S.P., Laprairie R.B., Wu Y., Qu L., Pu M., Korde A., Jiang S., Ho J.H. (2017). Crystal structures of agonist-bound human cannabinoid receptor CB1. Nature.

[25] Ryberg E., Vu H.K., Larsson N., Groblewski T., Hjorth S., Elebring T., Sjorgren S., Greasley P.J. (2005). Identification and characterisation of a novel splice variant of the human CB1receptor. FEBS Lett..

[26] Shire D., Carillon C., Kaghad M., Calandra B., Rinaldicarmona M., Lefur G., Caput D., Ferrara P. (1995). An amino-terminal variant of the central cannabinoid receptor resulting from alternative splicing. J. Biol. Chem..

[27] Gonzalez-Mariscal I., Krzysik-Walker S.M., Doyle M.E., Liu Q.R., Cimbro R., Calvo S.S.C., Ghosh S., Ciesla L., Moaddel R., Carlson O.D. (2016). Human CB1 receptor isoforms, present in hepatocytes and β-cells, are involved in regulating metabolism. Sci. Rep..

[28] Straiker A., Wager-Miller J., Hutchens J., Mackie K. (2012). Differential signalling in human cannabinoid CB1 receptors and their splice variants in autaptic hippocampal neurones. Br. J. Pharmacol..

[29] Xiao J.C., Jewell J.P., Lin L.S., Hagmann W.K., Fong T.M., Shen C.P. (2008). Similar in vitro pharmacology of human cannabinoid CB1 receptor variants expressed in cho cells. Brain Res..

[30] Zhang H.Y., Bi G.H., Li X., Li J., Qu H., Zhang S.J., Li C.Y., Onaivi E.S., Gardner E.L., Xi Z.X. (2015). Species differences in cannabinoid receptor 2 and receptor responses to cocaine self-administration in mice and rats. Neuropsychopharmacology.

[31] Liu Q.R., Pan C.H., Hishimoto A., Li C.Y., Xi Z.X., Llorente-Berzal A., Viveros M.P., Ishiguro H., Arinami T., Onaivi E.S. (2009). Species differences in cannabinoid receptor 2 (*CNR2* gene): Identification of novel human and rodent CB2 isoforms, differential tissue expression and regulation by cannabinoid receptor ligands. Genes Brain Behav..

[32] Di Marzo V., de Petrocellis L. (2012). Why do cannabinoid receptors have more than one endogenous ligand?. Philos. Trans. R. Soc. B.

[33] Castillo P.E., Younts T.J., Chavez A.E., Hashimotodani Y. (2012). Endocannabinoid signaling and synaptic function. Neuron.

[34] Katona I., Freund T.F. (2008). Endocannabinoid signaling as a synaptic circuit breaker in neurological disease. Nat. Med..

[35] Murataeva N., Straiker A., Mackie K. (2014). Parsing the players: 2-arachidonoylglycerol synthesis and degradation in the CNS. Br. J. Pharmacol..

[36] Huang H., McIntosh A.L., Martin G.G., Landrock D., Chung S., Landrock K.K., Dangott L.J., Li S.R., Kier A.B., Schroeder F. (2016). Fabp1: A novel hepatic endocannabinoid and cannabinoid binding protein. Biochemistry.

[37] Blankman J.L., Simon G.M., Cravatt B.F. (2007). A comprehensive profile of brain enzymes that hydrolyze the endocannabinoid 2-arachidonoylglycerol. Chem. Biol..

[38] Rouzer C.A., Marnett L.J. (2011). Endocannabinoid oxygenation by cyclooxygenases, lipoxygenases, and cytochromes p450: Cross-talk between the eicosanoid and endocannabinoid signaling pathways. Chem. Rev..

[39] Maccarrone M., Rossi S., Bari M., de Chiara V., Fezza F., Musella A., Gasperi V., Prosperetti C., Bernardi G., Finazzi-Agro A. (2008). Anandamide inhibits metabolism and physiological actions of 2-arachidonoylglycerol in the striatum. Nat. Neurosci..

[40] Ohno-Shosaku T., Kano M. (2014). Endocannabinoid-mediated retrograde modulation of synaptic transmission. Curr. Opin. Neurobiol..

[41] Khlaifia A., Farah H., Gackiere F., Tell F. (2013). Anandamide, cannabinoid type 1 receptor, and nmda receptor activation mediate non-hebbian presynaptically expressed long-term depression at the first central synapse for visceral afferent fibers. J. Neurosci..

[42] Puente N., Cui Y.H., Lassalle O., Lafourcade M., Georges F., Venance L., Grandes P., Manzoni O.J. (2011). Polymodal activation of the endocannabinoid system in the extended amygdala. Nat. Neurosci..

[43] Chavez A.E., Chiu C.Q., Castillo P.E. (2010). Trpv1 activation by endogenous anandamide triggers postsynaptic long-term depression in dentate gyrus. Nat. Neurosci..

[44] Grueter B.A., Brasnjo G., Malenka R.C. (2010). Postsynaptic trpv1 triggers cell type-specific long-term depression in the nucleus accumbens. Nat. Neurosci..

[45] Lerner T.N., Kreitzer A.C. (2012). Rgs4 is required for dopaminergic control of striatal ltd and susceptibility to parkinsonian motor deficits. Neuron.

[46] Schlosburg J.E., Blankman J.L., Long J.Z., Nomura D.K., Pan B., Kinsey S.G., Nguyen P.T., Ramesh D., Booker L., Burston J.J. (2010). Chronic monoacylglycerol lipase blockade causes functional antagonism of the endocannabinoid system. Nat. Neurosci..

[47] Marinelli S., Pacioni S., Bisogno T., di Marzo V., Prince D.A., Huguenard J.R., Bacci A. (2008). The endocannabinoid 2-arachidonoylglycerol is responsible for the slow self-inhibition in neocortical interneurons. J. Neurosci..

[48] Min R., Testa-Silva G., Heistek T.S., Canto C.B., Lodder J.C., Bisogno T., di Marzo V., Brussaard A.B., Burnashev N., Mansvelder H.D. (2010). Diacylglycerol lipase is not involved in depolarization-induced suppression of inhibition at unitary inhibitory connections in mouse hippocampus. J. Neurosci..

[49] Marinelli S., Pacioni S., Cannich A., Marsicano G., Bacci A. (2009). Self-modulation of neocortical pyramidal neurons by endocannabinoids. Nat. Neurosci..

[50] Bacci A., Huguenard J.R., Prince D.A. (2004). Long-lasting self-inhibition of neocortical interneurons mediated by endocannabinoids. Nature.

[51] Han J., Kesner P., Metna-Laurent M., Duan T.T., Xu L., Georges F., Koehl M., Abrous D.N., Mendizabal-Zubiaga J., Grandes P. (2012). Acute cannabinoids impair working memory through astroglial CB1 receptor modulation of hippocampal ltd. Cell.

[52] Navarrete M., Araque A. (2010). Endocannabinoids potentiate synaptic transmission through stimulation of astrocytes. Neuron.

[53] Navarrete M., Araque A. (2008). Endocannabinoids mediate neuron-astrocyte communication. Neuron.

[54] Stella N. (2009). Endocannabinoid signaling in microglial cells. Neuropharmacology.

[55] Dhopeshwarkar A., Mackie K. (2014). CB2 cannabinoid receptors as a therapeutic target-what does the future hold?. Mol. Pharmacol..

[56] Atwood B.K., Mackie K. (2010). CB_2_: A cannabinoid receptor with an identity crisis. Br. J. Pharmacol..

[57] Gong J.P., Onaivi E.S., Ishiguro H., Liu Q.R., Tagliaferro P.A., Brusco A., Uhl G.R. (2006). Cannabinoid CB2 receptors: Immunohistochemical localization in rat brain. Brain Res..

[58] Den Boon F.S., Chameau P., Schaafsma-Zhao Q., van Aken W., Bari M., Oddi S., Kruse C.G., Maccarrone M., Wadman W.J., Werkman T.R. (2012). Excitability of prefrontal cortical pyramidal neurons is modulated by activation of intracellular type-2 cannabinoid receptors. Proc. Natl. Acad. Sci. USA.

[59] Mackie K. (2005). Distribution of cannabinoid receptors in the central and peripheral nervous system. Handb. Exp. Pharmacol..

[60] Katona I., Sperlagh B., Sik A., Kafalvi A., Vizi E.S., Mackie K., Freund T.F. (1999). Presynaptically located CB1 cannabinoid receptors regulate GABA release from axon terminals of specific hippocampal interneurons. J. Neurosci..

[61] Tsou K., Brown S., Sanudo-Pena M.C., Mackie K., Walker J.M. (1998). Immunohistochemical distribution of cannabinoid CB1 receptors in the rat central nervous system. Neuroscience.

[62] Maroso M., Szabo G.G., Kim H.K., Alexander A., Bui A.D., Lee S.H., Lutz B., Soltesz I. (2016). Cannabinoid control of learning and memory through hcn channels. Neuron.

[63] Maccarrone M., Bab R., Biro T., Cabral G.A., Dey S.K., di Marzo V., Konje J.C., Kunos G., Mechoulam R., Pacher P. (2015). Endocannabinoid signaling at the periphery: 50 years after thc. Trends Pharmacol. Sci..

[64] Tam J., Trembovler V., di Marzo V., Petrosino S., Leo G., Alexandrovich A., Regev E., Casap N., Shteyer A., Ledent C. (2008). The cannabinoid CB1 receptor regulates bone formation by modulating adrenergic signaling. FASEB J..

[65] Clapper J.R., Moreno-Sanz G., Russo R., Guijarro A., Vacondio F., Duranti A., Tontini A., Sanchini S., Sciolino N.R., Spradley J.M. (2010). Anandamide suppresses pain initiation through a peripheral endocannabinoid mechanism. Nat. Neurosci..

[66] Price T.J., Helesic G., Parghi D., Hargreaves K.M., Flores C.M. (2003). The neuronal distribution of cannabinoid receptor type 1 in the trigeminal ganglion of the rat. Neuroscience.

[67] Veress G., Meszar Z., Muszil D., Avelino A., Matesz K., Mackie K., Nagy I. (2013). Characterisation of cannabinoid 1 receptor expression in the perikarya, and peripheral and spinal processes of primary sensory neurons. Brain Struct. Funct..

[68] Izzo A.A., Sharkey K.A. (2010). Cannabinoids and the gut: New developments and emerging concepts. Pharmacol. Ther..

[69] Miller L.K., Devi L.A. (2011). The highs and lows of cannabinoid receptor expression in disease: Mechanisms and their therapeutic implications. Pharmacol. Rev..

[70] Montecucco F., di Marzo V. (2012). At the heart of the matter: The endocannabinoid system in cardiovascular function and dysfunction. Trends Pharmacol. Sci..

[71] Rozenfeld R. (2011). Type I cannabinoid receptor trafficking: All roads lead to lysosome. Traffic.

[72] Leterrier C., Bonnard D., Carrel D., Rossier J., Lenkei Z. (2004). Constitutive endocytic cycle of the CB1 cannabinoid receptor. J. Biol. Chem..

[73] Grimsey N.L., Graham E.S., Dragunow M., Glass M. (2010). Cannabinoid receptor 1 trafficking and the role of the intracellular pool: Implications for therapeutics. Biochem. Pharmacol..

[74] Rozenfeld R., Devi L.A. (2008). Regulation of CB1 cannabinoid receptor trafficking by the adaptor protein ap-3. FASEB J..

[75] Brailoiu G.C., Oprea T.I., Zhao P., Abood M.E., Brailoiu E. (2011). Intracellular cannabinoid type 1 (CB_1_) receptors are activated by anandamide. J. Biol. Chem..

[76] Martin B.R. (1986). Cellular effects of cannabinoids. Pharmacol. Rev..

[77] Benard G., Massa F., Puente N., Lourenco J., Bellocchio L., Soria-Gomez E., Matias I., Delamarre A., Metna-Laurent M., Cannich A. (2012). Mitochondrial CB1 receptors regulate neuronal energy metabolism. Nat. Neurosci..

[78] Hebert-Chatelain E., Reguero L., Puente N., Lutz B., Chaouloff F., Rossignol R., Piazza P.V., Benard G., Grandes P., Marsicano G. (2014). Cannabinoid control of brain bioenergetics: Exploring the subcellular localization of the CB1 receptor. Mol. Metab..

[79] Hebert-Chatelain E., Reguero L., Puente N., Lutz B., Chaouloff F., Rossignol R., Piazza P.V., Benard G., Grandes P., Marsicano G. (2014). Studying mitochondrial CB1 receptors: Yes we can. Mol. Metab..

[80] Morozov Y.M., Horvath T.L., Rakic P. (2014). A tale of two methods: Identifying neuronal CB1 receptors. Mol. Metab..

[81] Koch M., Varela L., Kim J.G., Kim J.D., Hernandez-Nuno F., Simonds S.E., Castorena C.M., Vianna C.R., Elmquist J.K., Morozov Y.M. (2015). Hypothalamic pomc neurons promote cannabinoid-induced feeding. Nature.

[82] Ma L., Jia J., Niu W., Jiang T., Zhai Q., Yang L., Bai F., Wang Q., Xiong L. (2015). Mitochondrial CB1 receptor is involved in acea-induced protective effects on neurons and mitochondrial functions. Sci. Rep..

[83] Hebert-Chatelain E., Desprez T., Serrat R., Bellocchio L., Soria-Gomez E., Busquets-Garcia A., Pagano Zottola A.C., Delamarre A., Cannich A., Vincent P. (2016). A cannabinoid link between mitochondria and memory. Nature.

[84] Sheng Z.H., Cai Q. (2012). Mitochondrial transport in neurons: Impact on synaptic homeostasis and neurodegeneration. Nat. Rev. Neurosci..

[85] Mattson M.P., Gleichmann M., Cheng A. (2008). Mitochondria in neuroplasticity and neurological disorders. Neuron.

[86] Thibault K., Carrel D., Bonnard D., Gallatz K., Simon A., Biard M., Pezet S., Palkovits M., Lenkei Z. (2013). Activation-dependent subcellular distribution patterns of CB1 cannabinoid receptors in the rat forebrain. Cereb. Cortex.

[87] Brailoiu G.C., Deliu E., Marcu J., Hoffman N.E., Console-Bram L., Zhao P., Madesh M., Abood M.E., Brailoiu E. (2014). Differential activation of intracellular versus plasmalemmal CB2 cannabinoid receptors. Biochemistry.

[88] Demuth D.G., Molleman A. (2006). Cannabinoid signalling. Life Sci..

[89] Rhee M.H., Bayewitch M., Avidor-Reiss T., Levy R., Vogel Z. (1998). Cannabinoid receptor activation differentially regulates the various adenylyl cyclase isozymes. J. Neurochem..

[90] Maneuf Y.P., Brotchie J.M. (1997). Paradoxical action of the cannabinoid win 55,212-2 in stimulated and basal cyclic amp accumulation in rat globus pallidus slices. Br. J. Pharmacol..

[91] Glass M., Felder C.C. (1997). Concurrent stimulation of cannabinoid CB1 and dopamine d2 receptors augments camp accumulation in striatal neurons: Evidence for a gs linkage to the CB1 receptor. J. Neurosci..

[92] Bonhaus D.W., Chang L.K., Kwan J., Martin G.R. (1998). Dual activation and inhibition of adenylyl cyclase by cannabinoid receptor agonists: Evidence for agonist-specific trafficking of intracellular responses. J. Pharmacol. Exp. Ther..

[93] Lauckner J.E., Hille B., Mackie K. (2005). The cannabinoid agonist win55,212-2 increases intracellular calcium via CB1 receptor coupling to Gq/11 G proteins. Proc. Natl. Acad. Sci. USA.

[94] Turu G., Hunyady L. (2010). Signal transduction of the CB1 cannabinoid receptor. J. Mol. Endocrinol..

[95] Brown S.P., Safo P.K., Regehr W.G. (2004). Endocannabinoids inhibit transmission at granule cell to purkinje cell synapses by modulating three types of presynaptic calcium channels. J. Neurosci..

[96] Twitchell W., Brown S., Mackie K. (1997). Cannabinoids inhibit N- and P/Q-type calcium channels in cultured rat hippocampal neurons. J. Neurophysiol..

[97] Mackie K., Devane W.A., Hille B. (1993). Anandamide, an endogenous cannabinoid, inhibits calcium currents as a partial agonist in N18 neuroblastoma-cells. Mol. Pharmacol..

[98] Mackie K., Hille B. (1992). Cannabinoids inhibit N-type calcium channels in neuroblastoma glioma-cells. Proc. Natl. Acad. Sci. USA.

[99] Gergely G.S., Nora L., Noemi H., Tibor A., Zoltan N., Norbert H. (2014). Presynaptic calcium channel inhibition underlies CB1 cannabinoid receptor-mediated suppression of gaba release. J. Neurosci..

[100] Fisyunov A., Tsintsadze V., Min R., Burnashev N., Lozovaya N. (2006). Cannabinoids modulate the P-type high-voltage-activated calcium currents in purkinje neurons. J. Neurophysiol..

[101] Mackie K., Lai Y., Westenbroek R., Mitchell R. (1995). Cannabinoids activate an inwardly rectifying potassium conductance and inhibit Q-type calcium currents in att20 cells transfected with rat-brain cannabinoid receptor. J. Neurosci..

[102] Guo J., Ikeda S.R. (2004). Endocannabinoids modulate N-type calcium channels and G-protein-coupled inwardly rectifying potassium channels via CB1 cannabinoid receptors heterologously expressed in mammalian neurons. Mol. Pharmacol..

[103] Robbe D., Alonso G., Duchamp F., Bockaert J., Manzoni O.J. (2001). Localization and mechanisms of action of cannabinoid receptors at the glutamatergic synapses of the mouse nucleus accumbens. J. Neurosci..

[104] Howlett A.C., Blume L.C., Dalton G.D. (2010). CB1 cannabinoid receptors and their associated proteins. Curr. Med. Chem..

[105] Galve-Roperh I., Rueda D., del Pulgar T.G., Velasco G., Guzman M. (2002). Mechanism of extracellular signal-regulated kinase activation by the CB1 cannabinoid receptor. Mol. Pharmacol..

[106] Flores-Otero J., Ahn K.H., Delgado-Peraza F., Mackie K., Kendall D.A., Yudowski G.A. (2014). Ligand-specific endocytic dwell times control functional selectivity of the cannabinoid receptor 1. Nat. Commun..

[107] Bouaboula M., Poinotchazel C., Bourrie B., Canat X., Calandra B., Rinaldicarmona M., Lefur G., Casellas P. (1995). Activation of mitogen-activated protein-kinases by stimulation of the central cannabinoid receptor CB1. Biochem. J..

[108] Derkinderen P., Ledent C., Parmentier M., Girault J.A. (2001). Cannabinoids activate p38 mitogen-activated protein kinases through CB1 receptors in hippocampus. J. Neurochem..

[109] Rueda D., Galve-Roperh I., Haro A., Guzman M. (2000). The CB1 cannabinoid receptor is coupled to the activation of c-jun N-terminal kinase. Mol. Pharmacol..

[110] Liu J., Gao B., Mirshahi F., Sanyal A.J., Khanolkar A.D., Makriyannis A., Kunos G. (2000). Functional CB1 cannabinoid receptors in human vascular endothelial cells. Biochem. J..

[111] He J.C.J., Gomes I., Nguyen T., Jayaram G., Ram P.T., Devi L.A., Iyengar R. (2005). The Gα_o/i_-coupled cannabinoid receptor-mediated neurite outgrowth involves rap regulation of src and stat3. J. Biol. Chem..

[112] McCudden C.R., Hains M.D., Kimple R.J., Siderovski D.P., Willard F.S. (2005). G-protein signaling: Back to the future. Cell. Mol. Life Sci..

[113] Kouznetsova M., Kelley B., Shen M.X., Thayer S.A. (2002). Desensitization of cannabinoid-mediated presynaptic inhibition of neurotransmission between rat hippocampal neurons in culture. Mol. Pharmacol..

[114] Jin W.Z., Brown S., Roche J.P., Hsieh C., Celver J.P., Kovoor A., Chavkin C., Mackie K. (1999). Distinct domains of the CB1 cannabinoid receptor mediate desensitization and internalization. J. Neurosci..

[115] Daigle T.L., Kearn C.S., Mackie K. (2008). Rapid CB1 cannabinoid receptor desensitization defines the time course of erk1/2 map kinase signaling. Neuropharmacology.

[116] Nguyen P.T., Schmid C.L., Raehal K.M., Selley D.E., Bohn L.M., Sim-Selley L.J. (2012). Beta-arrestin2 regulates cannabinoid CB1 receptor signaling and adaptation in a central nervous system region-dependent manner. Biol. Psychiatry.

[117] Breivogel C.S., Lambert J.M., Gerfin S., Huffman J.W., Razdan R.K. (2008). Sensitivity to delta 9-tetrahydrocannabinol is selectively enhanced in beta-arrestin2^−/−^ mice. Behav. Pharmacol..

[118] Ahn K.H., Mahmoud M.M., Shim J.Y., Kendall D.A. (2013). Distinct roles of beta-arrestin 1 and beta-arrestin 2 in org27569-induced biased signaling and internalization of the cannabinoid receptor 1 (CB1). J. Biol. Chem..

[119] Gomez del Pulgar T., Velasco G., Guzman M. (2000). The CB1 cannabinoid receptor is coupled to the activation of protein kinase B/Akt. Biochem. J..

[120] Gomez O., Sanchez-Rodriguez A., Le M.Q.U., Sanchez-Caro C., Molina-Holgado F., Molina-Holgado E. (2011). Cannabinoid receptor agonists modulate oligodendrocyte differentiation by activating pi3k/akt and the mammalian target of rapamycin (mtor) pathways. Br. J. Pharmacol..

[121] Molina-Holgado E., Vela J.M., Arevalo-Martin A., Almazan G., Molina-Holgado F., Borrell J., Guaza C. (2002). Cannabinoids promote oligodendrocyte progenitor survival: Involvement of cannabinoid receptors and phosphatidylinositol-3 kinase/akt signaling. J. Neurosci..

[122] Molina-Holgado F., Pinteaux E., Heenan L., Moore J.D., Rothwell N.J., Gibson R.M. (2005). Neuroprotective effects of the synthetic cannabinoid hu-210 in primary cortical neurons are mediated by phosphatidylinositol 3-kinase/akt signaling. Mol. Cell. Neurosci..

[123] Ozaita A., Puighermanal E., Maldonado R. (2007). Regulation of pi3k/akt/gsk-3 pathway by cannabinoids in the brain. J. Neurochem..

[124] Blazquez C., Chiarlone A., Bellocchio L., Resel E., Pruunsild P., Garcia-Rincon D., Sendtner M., Timmusk T., Lutz B., Galve-Roperh I. (2015). The CB1 cannabinoid receptor signals striatal neuroprotection via a pi3k/akt/mtorc1/bdnf pathway. Cell. Death Differ..

[125] Lopez-Cardona A.P., Perez-Cerezales S., Fernandez-Gonzalez R., Laguna-Barraza R., Pericuesta E., Agirregoitia N., Gutierrez-Adan A., Agirregoitia E. (2017). CB1 cannabinoid receptor drives oocyte maturation and embryo development via pi3k/akt and mapk pathways. FASEB J..

[126] Di Marzo V., Stella N., Zimmer A. (2015). Endocannabinoid signalling and the deteriorating brain. Nat. Rev. Neurosci..

[127] Iversen L. (2003). Cannabis and the brain. Brain.

[128] Di Marzo V. (2008). Targeting the endocannabinoid system: To enhance or reduce?. Nat. Rev. Drug Discov..

[129] Gerdeman G., Lovinger D.M. (2001). CB1 cannabinoid receptor inhibits synaptic release of glutamate in rat dorsolateral striatum. J. Neurophysiol..

[130] Chiarlone A., Bellocchio L., Blazquez C., Resel E., Soria-Gomez E., Cannich A., Ferrero J.J., Sagredo O., Benito C., Romero J. (2014). A restricted population of CB1 cannabinoid receptors with neuroprotective activity. Proc. Natl. Acad Sci. USA.

[131] Marsicano G., Goodenough S., Monory K., Hermann H., Eder M., Cannich A., Azad S.C., Cascio M.G., Gutierrez S.O., van der Stelt M. (2003). CB1 cannabinoid receptors and on-demand defense against excitotoxicity. Science.

[132] Zoppi S., Nievas B.G.P., Madrigal J.L.M., Manzanares J., Leza J.C., Garcia-Bueno B. (2011). Regulatory role of cannabinoid receptor 1 in stress-induced excitotoxicity and neuroinflammation. Neuropsychopharmacology.

[133] Blazquez C., Chiarlone A., Sagredo O., Aguado T., Pazos M.R., Resel E., Palazuelos J., Julien B., Salazar M., Borner C. (2011). Loss of striatal type 1 cannabinoid receptors is a key pathogenic factor in huntington’s disease. Brain.

[134] Kim S.H., Won S.J., Mao X.O., Jin K., Greenberg D.A. (2006). Molecular mechanisms of cannabinoid protection from neuronal excitotoxicity. Mol. Pharmacol..

[135] Khaspekov L.G., Verca M.S.B., Frumkina L.E., Hermann H., Marsicano G., Lutz B. (2004). Involvement of brain-derived neurotrophic factor in cannabinoid receptor-dependent protection against excitotoxicity. Eur. J. Neurosci..

[136] Sanchez-Blazquez P., Rodriguez-Munoz M., Vicente-Sanchez A., Garzon J. (2013). Cannabinoid receptors couple to nmda receptors to reduce the production of no and the mobilization of zinc induced by glutamate. Antioxid. Redox Signal..

[137] Vicente-Sanchez A., Sanchez-Blazquez P., Rodriguez-Munoz M., Garzon J. (2013). Hint1 protein cooperates with cannabinoid 1 receptor to negatively regulate glutamate nmda receptor activity. Mol. Brain.

[138] Brotchie J.M. (2003). CB1 cannabinoid receptor signalling in parkinson’s disease. Curr. Opin. Pharmacol..

[139] Waksman Y., Olson J.M., Carlisle S.J., Cabral G.A. (1999). The central cannabinoid receptor (CB1) mediates inhibition of nitric oxide production by rat microglial cells. J. Pharmacol. Exp. Ther..

[140] Milton N.G.N. (2002). Anandamide and noladin ether prevent neurotoxicity of the human amyloid-beta peptide. Neurosci. Lett..

[141] Benito C., Nunez E., Tolon R.M., Carrier E.J., Rabano A., Hillard C.J., Romero J. (2003). Cannabinoid CB2 receptors and fatty acid amide hydrolase are selectively overexpressed in neuritic plaque-associated glia in alzheimer’s disease brains. J. Neurosci..

[142] Romero J., Berrendero F., Garcia-Gil L., de la Cruz P., Ramos J.A., Fernandez-Ruiz J.J. (1998). Loss of cannabinoid receptor binding and messenger RNA levels and cannabinoid agonist-stimulated [^35^S]guanylyl-5′-*O*-(thio)-triphosphate binding in the basal ganglia of aged rats. Neuroscience.

[143] Westlake T.M., Howlett A.C., Bonner T.I., Matsuda L.A., Herkenham M. (1994). Cannabinoid receptor-binding and messenger-RNA expression in human brain—An in-vitro receptor autoradiography and in-situ hybridization histochemistry study of normal aged and alzheimers brains. Neuroscience.

[144] Ramirez B.G., Blazquez C., Gomez del Pulgar T., Guzman M., de Ceballos M.L. (2005). Prevention of alzheimer’s disease pathology by cannabinoids: Neuroprotection mediated by blockade of microglial activation. J. Neurosci..

[145] Haghani M., Shabani M., Javan M., Motamedi F., Janahmadi M. (2012). CB1 cannabinoid receptor activation rescues amyloid beta-induced alterations in behaviour and intrinsic electrophysiological properties of rat hippocampal ca1 pyramidal neurones. Cell. Physiol. Biochem..

[146] Aso E., Palomer E., Juves S., Maldonado R., Munoz F.J., Ferrer I. (2012). CB1 agonist acea protects neurons and reduces the cognitive impairment of AβPP/PS1 mice. J. Alzheimers Dis..

[147] Van der Stelt M., Mazzola C., Esposito G., Matias I., Petrosino S., de Filippis D., Micale V., Steardo L., Drago F., Iuvone T. (2006). Endocannabinoids and beta-amyloid-induced neurotoxicity in vivo: Effect of pharmacological elevation of endocannabinoid levels. Cell. Mol. Life Sci..

[148] Glass M., Faull R.L.M., Dragunow M. (1993). Loss of cannabinoid receptors in the substantia-nigra in huntingtons-disease. Neuroscience.

[149] Glass M., Dragunow M., Faull R.L.M. (2000). The pattern of neurodegeneration in huntington’s disease: A comparative study of cannabinoid, dopamine, adenosine and GABA_A_ receptor alterations in the human basal ganglia in huntington’s disease. Neuroscience.

[150] Horne E.A., Coy J., Swinney K., Fung S., Cherry A.E.T., Marrs W.R., Naydenov A.V., Lin Y.H., Sun X.C., Keene C.D. (2013). Downregulation of cannabinoid receptor 1 from neuropeptide y interneurons in the basal ganglia of patients with huntington’s disease and mouse models. Eur. J. Neurosci..

[151] Glass M., Van Dellen A., Blakemore C., Hannan A.J., Faull R.L.M. (2004). Delayed onset of huntington’s disease in mice in an enriched environment correlates with delayed loss of cannabinoid CB1 receptors. Neuroscience.

[152] Mievis S., Blum D., Ledent C. (2011). Worsening of huntington disease phenotype in CB1 receptor knockout mice. Neurobiol. Dis..

[153] Naydenov A.V., Sepers M.D., Swinney K., Raymond L.A., Palmiter R.D., Stella N. (2014). Genetic rescue of CB1 receptors on medium spiny neurons prevents loss of excitatory striatal synapses but not motor impairment in hd mice. Neurobiol. Dis..

[154] Dowie M.J., Howard M.L., Nicholson L.F.B., Faull R.L.M., Hannan A.J., Glass M. (2010). Behavioural and molecular consequences of chronic cannabinoid treatment in huntington’s disease transgenic mice. Neuroscience.

[155] Ellison J.M., Gelwan E., Ogletree J. (1990). Complex partial seizure symptoms affected by marijuana abuse. J. Clin. Psychiatry.

[156] Consroe P.F., Wood G.C., Buchsbaum H. (1975). Anticonvulsant nature of marihuana smoking. JAMA.

[157] Keeler M.H., Reifler C.B. (1967). Grand mal convulsions subsequent to marijuana use—Case report. Dis. Nerv. Syst..

[158] Clement A.B., Hawkins E.G., Lichtman A.H., Cravatt B.F. (2003). Increased seizure susceptibility and proconvulsant activity of anandamide in mice lacking fatty acid amide hydrolase. J. Neurosci..

[159] Wallace M.J., Martin B.R., DeLorenzo R.J. (2002). Evidence for a physiological role of endocannabinoids in the modulation of seizure threshold and severity. Eur. J. Pharmacol..

[160] Chen K., Neu A., Howard A.L., Foldy C., Echegoyen J., Hilgenberg L., Smith M., Mackie K., Soltesz I. (2007). Prevention of plasticity of endocannabinoid signaling inhibits persistent limbic hyperexcitability caused by developmental seizures. J. Neurosci..

[161] Chen K., Ratzliff A., Hilgenberg L., Gulyas A., Freund T.F., Smith M., Dinh T.P., Piomelli D., Mackie K., Soltesz I. (2003). Long-term plasticity of endocannabinoid signaling induced by developmental febrile seizures. Neuron.

[162] Bhaskaran M.D., Smith B.N. (2010). Cannabinoid-mediated inhibition of recurrent excitatory circuitry in the dentate gyrus in a mouse model of temporal lobe epilepsy. PLoS ONE.

[163] Falenski K.W., Blair R.E., Sim-Selley L.J., Martin B.R., DeLorenzo R.J. (2007). Status epilepticus causes a long-lasting redistribution of hippocampal cannabinoid type 1 receptor expression and function in the rat pilocarpine model of acquired epilepsy. Neuroscience.

[164] Wallace M.J., Blair R.E., Falenski K.W., Martin B.R., DeLorenzo R.J. (2003). The endogenous cannabinoid system regulates seizure frequency and duration in a model of temporal lobe epilepsy. J. Pharmacol. Exp. Ther..

[165] Falenski K.W., Carter D.S., Harrison A.J., Martin B.R., Blair R.E., DeLorenzo R.J. (2009). Temporal characterization of changes in hippocampal cannabinoid CB1 receptor expression following pilocarpine-induced status epilepticus. Brain Res..

[166] Di Marzo V., Matias I. (2005). Endocannabinoid control of food intake and energy balance. Nat. Neurosci..

[167] Kirkham T.C., Williams C.M., Fezza F., di Marzo V. (2002). Endocannabinoid levels in rat limbic forebrain and hypothalamus in relation to fasting, feeding and satiation: Stimulation of eating by 2-arachidonoyl glycerol. Br. J. Pharmacol..

[168] Bellocchio L., Lafenetre P., Cannich A., Cota D., Puente N., Grandes P., Chaouloff F., Piazza P.V., Marsicano G. (2010). Bimodal control of stimulated food intake by the endocannabinoid system. Nat. Neurosci..

[169] Soria-Gomez E., Bellocchio L., Reguero L., Lepousez G., Martin C., Bendahmane M., Ruehle S., Remmers F., Desprez T., Matias I. (2014). The endocannabinoid system controls food intake via olfactory processes. Nat. Neurosci..

[170] Moreira F.A., Crippa J.A. (2009). The psychiatric side-effects of rimonabant. Rev. Bras. Psiquiatr..

[171] Koch M. (2017). Cannabinoid receptor signaling in central regulation of feeding behavior: A mini-review. Front. Neurosci..

[172] Fine P.G., Rosenfeld M.J. (2013). The endocannabinoid system, cannabinoids, and pain. Rambam Maimonides Med. J..

[173] Donvito G., Nass S.R., Wilkerson J.L., Curry Z.A., Schurman L.D., Kinsey S.G., Lichtman A.H. (2017). The endogenous cannabinoid system: A budding source of targets for treating inflammatory and neuropathic pain. Neuropsychopharmacology.

[174] Akopian A.N., Ruparel N.B., Jeske N.A., Patwardhan A., Hargreaves K.M. (2009). Role of ionotropic cannabinoid receptors in peripheral antinociception and antihyperalgesia. Trends Pharmacol. Sci..

[175] Jhaveri M.D., Sagar D.R., Elmes S.J.R., Kendall D.A., Chapman V. (2007). Cannabinoid CB2 receptor-mediated anti-nociception in models of acute and chronic pain. Mol. Neurobiol..

[176] Russo E.B. (2008). Cannabinoids in the management of difficult to treat pain. Ther. Clin. Risk Manag..

[177] Laprairie R.B., Bagher A.M., Kelly M.E., Denovan-Wright E.M. (2015). Cannabidiol is a negative allosteric modulator of the cannabinoid CB1 receptor. Br. J. Pharmacol..

[178] Hall W., Christie M.J., Currow D. (2005). Cannabinoids and cancer: Causation, remediation, and palliation. Lancet Oncol..

[179] Guzman M. (2003). Cannabinoids: Potential anticancer agents. Nat. Rev. Cancer.

[180] Velasco G., Sanchez C., Guzman M. (2012). Towards the use of cannabinoids as antitumour agents. Nat. Rev. Cancer.

[181] Pisanti S., Picardi P., D’Alessandro A., Laezza C., Bifulco M. (2013). The endocannabinoid signaling system in cancer. Trends Pharmacol. Sci..

[182] Sanchez C., de Ceballos M.L., del Pulgar T.G., Rueda D., Corbacho C., Velasco G., Galve-Roperh I., Huffman J.W., Cajal S.R.Y., Guzman M. (2001). Inhibition of glioma growth in vivo by selective activation of the CB2 cannabinoid receptor. Cancer Res..

[183] Caffarel M.M., Sarrio D., Palacios J., Guzman M., Sanchez C. (2006). Δ^9^-tetrahydrocannabinol inhibits cell cycle progression in human breast cancer cells through cdc2 regulation. Cancer Res..

[184] Hart S., Fischer O.M., Ullrich A. (2004). Cannabinoids induce cancer cell proliferation via tumor necrosis factor alpha-converting enzyme (tace/adam17)-mediated transactivation of the epidermal growth factor receptor. Cancer Res..

[185] Abramowicz M., Zuccotti G., Pflomm J.M. (2016). Cannabis and cannabinoids. JAMA.

[186] Volkow N.D., Swanson J.M., Evins A.E., DeLisi L.E., Meier M.H., Gonzalez R., Bloomfield M.A.P., Curran H.V., Baler R. (2016). Effects of cannabis use on human behavior, including cognition, motivation, and psychosis: A review. JAMA Psychiatry.

[187] Bauer M., Chicca A., Tamborrini M., Eisen D., Lerner R., Lutz B., Poetz O., Pluschke G., Gertsch J. (2012). Identification and quantification of a new family of peptide endocannabinoids (pepcans) showing negative allosteric modulation at CB1 receptors. J. Biol. Chem..

[188] Pamplona F.A., Ferreira J., Menezes de Lima O., Duarte F.S., Bento A.F., Forner S., Villarinho J.G., Bellocchio L., Wotjak C.T., Lerner R. (2012). Anti-inflammatory lipoxin a4 is an endogenous allosteric enhancer of CB1 cannabinoid receptor. Proc. Natl. Acad. Sci. USA.

[189] Ignatowska-Jankowska B.M., Baillie G.L., Kinsey S., Crowe M., Ghosh S., Owens R.A., Damaj I.M., Poklis J., Wiley J.L., Zanda M. (2015). A cannabinoid CB1 receptor-positive allosteric modulator reduces neuropathic pain in the mouse with no psychoactive effects. Neuropsychopharmacology.

[190] Khurana L., Mackie K., Piomelli D., Kendall D.A. (2017). Modulation of CB1 cannabinoid receptor by allosteric ligands: Pharmacology and therapeutic opportunities. Neuropharmacology.

[191] Hudson B.D., Hebert T.E., Kelly M.E.M. (2010). Ligand- and heterodimer-directed signaling of the CB1 cannabinoid receptor. Mol. Pharmacol..

[192] Fujita W., Gomes I., Dove L.S., Prohaska D., McIntyre G., Devi L.A. (2014). Molecular characterization of eluxadoline as a potential ligand targeting mu-delta opioid receptor heteromers. Biochem. Pharmacol..

[193] Keating G.M. (2017). Eluxadoline: A review in diarrhoea-predominant irritable bowel syndrome. Drugs.

[194] Culler M.D. (2011). Somatostatin-dopamine chimeras: A novel approach to treatment of neuroendocrine tumors. Horm Metab. Res..

[195] Perrey D.A., Gilmour B.P., Thomas B.F., Zhang Y.A. (2014). Toward the development of bivalent ligand probes of cannabinoid CB1 and orexin ox1 receptor heterodimers. ACS Med. Chem. Lett..

[196] Glass M., Govindpani K., Furkert D.P., Hurst D.P., Reggio P.H., Flanagan J.U. (2016). One for the price of two...Are bivalent ligands targeting cannabinoid receptor dimers capable of simultaneously binding to both receptors?. Trends Pharmacol. Sci..

[197] Van Esbroeck A.C.M., Janssen A.P.A., Cognetta A.B., Ogasawara D., Shpak G., van der Kroeg M., Kantae V., Baggelaar M.P., de Vrij F.M.S., Deng H. (2017). Activity-based protein profiling reveals off-target proteins of the faah inhibitor bia 10-2474. Science.

